# An Integrated Transcriptomic and Proteomic Approach Uncovers the Molecular Mechanisms of Hypoosmotic Adaptation in *Scylla paramamosain* Megalopa

**DOI:** 10.3390/ijms26189188

**Published:** 2025-09-20

**Authors:** Ning Qiao, Zhiqiang Liu, Yuanyuan Li, Fengying Zhang, Chunyan Ma, Xueyang Wang, Jiayuan Xu, Lingbo Ma, Keyi Ma, Wei Wang

**Affiliations:** 1 Key Laboratory of East China Sea Fishery Resources Exploitation, Ministry of Agriculture and Rural Affairs, East China Sea Fisheries Research Institute, Chinese Academy of Fishery Sciences, Shanghai 200090, China; 2 Ninghai Fishery Innovation Research Center, Ningbo 315604, China; 3 Rizhao Ocean and Fishery Research Institute, Rizhao 276800, China

**Keywords:** *Scylla paramamosain*, megalopa, salinity stress, transcriptomics, proteomics

## Abstract

Salinity is a pivotal environmental factor that governs crustacean survival and development through its regulatory effects on key physiological processes, including osmoregulation and metabolic homeostasis. In the mud crab *Scylla paramamosain*, salinity tolerance of the megalopa plays an important role in larval survival rates and aquaculture yield. Here, we employed a combined transcriptomic and proteomic strategy to comprehensively dissect the molecular adaptive mechanisms of *S. paramamosain* megalopa exposed to acute and prolonged low-salinity stress (8‰) compared to control condition (17‰). Illumina-based transcriptome sequencing generated 81.71 Gb of high-quality clean data, which were assembled into 42,210 unigenes. LC-MS/MS-based proteomic profiling identified 51,390 unique peptides, corresponding to 5909 confidently quantified proteins. Transcriptomic profiling identified 2627 differentially expressed genes (DEGs) under acute low-salinity stress, comprising 1332 upregulated and 1295 downregulated genes compared to the control group. In contrast, a total of 733 DEGs were identified under prolonged low-salinity exposure, including 390 upregulated and 343 downregulated genes. Parallel proteomic analysis identified 199 differentially expressed proteins (DEPs) in the acute stress group, with 105 upregulated and 94 downregulated relative to the control group. Under prolonged stress, 206 DEPs were detected, including 124 upregulated and 82 downregulated proteins compared to the control group. Significant GO term and KEGG pathway enrichments contained metal ion binding, oxidoreductase activity, nucleus, apoptotic process, innate immune response, and amino acid metabolism, suggesting that megalopa employ coordinated regulatory mechanisms involving metabolic reprogramming, immunity system modulation, ion homeostasis maintenance and cell cycle regulation to adapt to hypoosmotic stress. Integrated multi-omics analysis identified 17 genes displaying significant concordant differential expression at both mRNA and protein levels during acute hypoosmotic stress, versus only 5 gene-protein pairs during prolonged stress exposure, indicating extensive post-transcriptional regulation and protein turnover mechanisms in sustained hypoosmotic condition. To the best of our knowledge, this study established the first integrative transcriptome-proteome framework elucidating hypoosmotic adaptation (8‰) mechanisms in *S. paramamosain* megalopa. The identified molecular signatures offer actionable targets for selective breeding of salinity-tolerant strains and precision management of megalopa culture under suboptimal salinity condition, while fundamentally advancing our mechanistic understanding of osmoregulatory plasticity across decapod crustaceans.

## 1. Introduction

Salinity serves as a key abiotic factor that modulates physiological processes, determines spatial distribution, and regulates population dynamics in euryhaline species, with particularly significant impacts on decapod crustaceans. Estuarine habitats experience dynamic salinity fluctuations driven by tidal cycles, precipitation regimes and fluvial inputs, creating substantial osmoregulatory challenges for resident species [1,2]. Early developmental stages of *Scylla paramamosain* (particularly zoea to megalopa transitions) exhibit heightened osmo-sensitivity, where deviations from species-specific salinity optima can disrupt metamorphic progression and significantly reduce survival rate [3].

Crustaceans maintain osmotic homeostasis in fluctuating salinity environments through sophisticated hyper-osmoregulatory mechanisms, with specialized ion transport systems localized in gill epithelia and antennal glands [4,5]. Key osmoregulatory proteins including the Na^+^/K^+^-ATPase, carbonic anhydrase and aquaporins orchestrate transcellular ion flux and water exchange, enabling rapid acclimation to osmotic challenges [6,7]. Concurrently, adaptive metabolic responses, including amino acid catabolism and enhanced energy metabolism, provide necessary energy reserves to sustain active osmoregulation and mitigate cellular stress induced by hypoosmotic exposure [8,9]. It is proposed that some salinity adaptation strategies are accomplished via a suite of transporters and enzymes, and supported by enhanced energy metabolism (e.g., carbohydrate and amino acid catabolism) that provides ATP to sustain active ion transports, forming an integrated osmoregulatory network. For example, the Na^+^ uptake pathway involves apical Na^+^ channels functionally coupled with V-type H^+^ ATPase proton pumps, complemented by basolateral Na^+^/K^+^ ATPase, while Cl^−^ absorption occurs through apical Na^+^-K^+^-2Cl^−^ co-transporters [10]. Although crustaceans exhibit broad salinity tolerance, rapid salinity variations beyond critical thresholds, could induce significant stress responses. Gene expression modulation is recognized as a central mechanism in stress adaptation, with rapid induction of stress-responsive genes preceding physiological adjustments. Prior studies indicated that numerous genes in mud crabs were involved in osmoregulation processes, such as V-type proton ATPase genes in ion transport, mitogen-activated protein kinase genes in signal transduction, NADH dehydrogenase-related genes in energy metabolism and heat shock protein genes in stress response [9,11].

The advent of high-throughput omics platforms, particularly next-generation RNA sequencing and mass spectrometry-based proteomics, has revolutionized our mechanistic understanding of osmoregulatory adaptation in euryhaline crustaceans [3,12]. Transcriptomic profiling has systematically revealed extensive changes in gene expression characteristics associated with ion regulation, metabolic reprogramming and stress response under salinity fluctuation [8,13]. Quantitative proteomics further complements transcriptomic insights, identifying proteins actively engaged in ion transport, metabolism, and stress responses, although disparities between transcript and protein expression patterns frequently occur due to post-transcriptional regulation [3,9]. Recent investigations focusing on *S. paramamosain* have demonstrated distinct molecular responses at both transcriptional and translational levels when exposed to osmotic stress. Notably, hyposaline exposure significantly induced the expression of ion transport-related genes and enhanced energy metabolism pathways, whereas hypersaline environments primarily triggered amino acid metabolism-related processes [9]. Nevertheless, systematic integration of transcriptomic and proteomic data during the megalopa stage remains scarce, particularly regarding post-transcriptional regulatory mechanisms.

**Figure 1:**
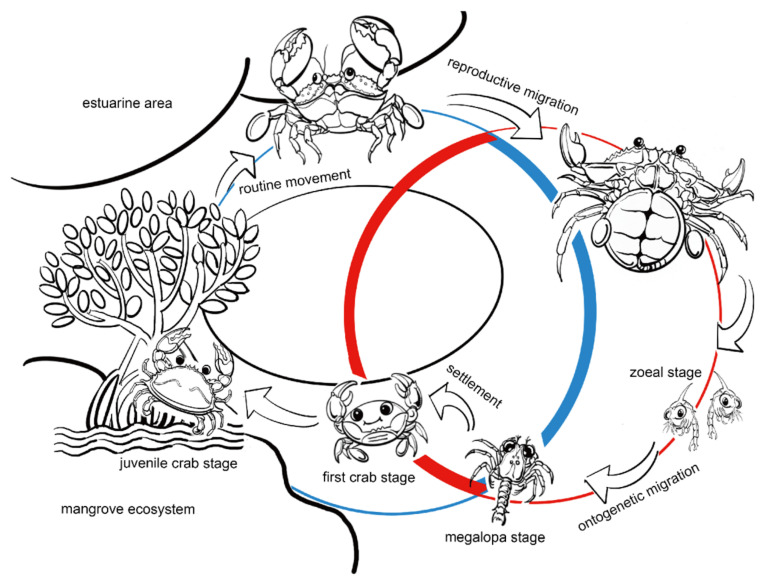
The life cycle of *S. paramamosain.* The schematic was originally drawn by the authors based on published descriptions of developmental stages and ecological migration patterns.

The mud crab *S. paramamosain* represents an economically important aquaculture species widely distributed across the Indo-West Pacific region, prized for its exceptional growth performance and substantial commercial value [14,15,16]. As a euryhaline organism, *S. paramamosain* inhabits waters of salinity around 5~33‰ [17], making it an ideal model for studying osmoregulation in brachyuran crabs [9]. However, rapid salinity fluctuations can cause severe physiological stress and elevated mortality rates, particularly during sensitive ontogenetic stages [11]. Exhibiting a biphasic life cycle strategy, *S. paramamosain* undergoes distinct ontogenetic habitat shifts between offshore marine and inshore estuarine environments (Figure 1). Zoeal stages primarily develop in high-salinity marine waters, reflecting an oceanic spawning adaptation. During the zoea-to-megalopa transition, larvae migrate toward estuarine nursery habitats, experiencing dramatic salinity decreases that challenge their osmoregulatory systems [18]. Following subsequent molting, megalopae metamorphose into juvenile crabs capable of inhabiting even lower salinity environments. After undergoing several molting cycles, mature individuals return to the higher salinity of the ocean for reproduction. The megalopa stage represents a critical developmental transition in *S. paramamosain*, characterized by substantial physiological adaptations during the high-to-low salinity habitat shift. Given the extended transition period and high mortality rates observed during commercial larval rearing of *S. paramamosain*, investigating megalopa osmoregulatory mechanisms is essential for enhancing survival rates and optimizing aquaculture production [11,15,16,19,20].

Although numerous studies have investigated osmoregulation in *S. paramamosain* [8,9,11], comprehensive understanding of systematic osmoregulatory mechanisms during the megalopa stage remains limited [11]. By what molecular mechanisms does the megalopa of *S. paramamosain* mediate osmotic regulation in response to fluctuating salinity conditions? To address this knowledge gap, *S. paramamosain* megalopae were collected and exposed to low-salinity condition (8‰) versus control condition (17‰) for acute (2 h) and prolonged (72 h) trials in the present study. Integrated RNA sequencing (RNA-seq) and data-independent acquisition (DIA) proteomics were employed to characterize differentially expressed genes (DEGs) and proteins (DEPs), revealing key molecular pathways governing osmoregulatory plasticity. Our findings provided valuable insights into salinity adaptive responses at the molecular level for *S. paramamosain* megalopa, contributing significantly to the enhancement of larval survival strategies and the optimization of aquaculture practices.

## 2. Results

### 2.1. Overview of Transcriptome and Proteome Sequencing and Assembly

Transcriptome sequencing generated high-quality data with 43.42–47.26 million clean paired-end reads per sample, exhibiting Q20 > 96.75% and Q30 > 92.5% (Table 1), meeting established standards for downstream analyses (Table 1). GC content remained stable among replicates (52.28–53.89%), with transcript length distribution peaking at 200–600 bp, indicating a dominance of short-length transcripts. *De novo* assembly produced 42,210 non-redundant unigenes with N50 of 2194 bp. BLAST searches against NCBI nr database annotated 21,944 unigenes (52.0% of total), while 11,561 unigenes (27.4%) received Gene Ontology (GO) terms, representing comprehensive functional coverage. Additionally, Kyoto Encyclopedia of Genes and Genomes (KEGG) pathway analysis identified enrichment in 138 signaling pathways.

DIA proteomic profiling identified a total of 51,390 unique peptides, corresponding to 5909 high-confidence proteins at a 1% FDR, with the majority (72.3%) of protein identifications supported by one or two unique peptides. Notably, a total of 1616 proteins (27.3%) were supported by over 10 peptides, representing key structural or functional proteins. Molecular weight distribution analysis revealed 262 low-mass proteins (<10 kD), 5063 proteins (85.7%) in the 10–100 kD range, 490 proteins (8.3%) between 100–200 kD and 86 high-molecular-weight proteins (>200 kD, 1.46%).

### 2.2. Principal Component Analysis (PCA)

**Figure 2:**
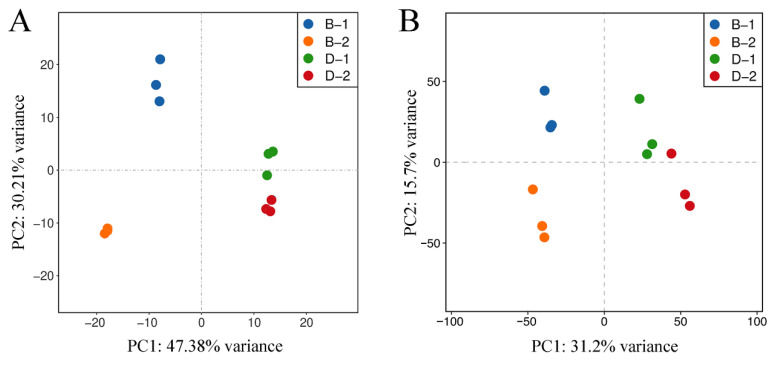
Principal component analysis (PCA) score plots of transcriptomic (**A**) and proteomic (**B**) datasets from *S. paramamosain* megalopa under salinity stress. B-1, acute stress (2 h, 8‰); B-2, acute control (2 h, 17‰); D-1, chronic stress (72 h, 8‰); and D-2, chronic control (72 h, 17‰).

PCA demonstrated clear segregation among the four groups along the principal coordinates (Figure 2). Transcriptomic PCA exhibited tight clustering of biological replicates within each treatment group (B-1: acute stress; B-2: acute control; D-1: prolonged stress; and D-2: prolonged control) (Figure 2A). The first two principal components (PC1 and PC2) collectively explained 77.59% of total variance (PC1: 47.38%; PC2: 30.21%), indicating strong discriminatory power. The results reflected that the transcriptomic profiles of different treatment groups were well separated, reflecting high data reproducibility and reliable sequencing quality.

Proteomic PCA showed more moderate but still significant variance explanation (PC1: 31.2%; PC2: 15.7%; Figure 2B), suggesting that proteomic responses to salinity stress could be partially distinguished among treatment groups. Notably, acute stress samples displayed the most homogeneous clustering pattern, suggesting highly consistent proteomic responses to acute hypoosmotic challenge. The close clustering of intra-group replicates confirmed high proteomic data quality, meeting stringent requirements for the following differential expression analysis.

### 2.3. Differential Expression Analysis of Transcriptomic and Proteomic Data

**Figure 3:**
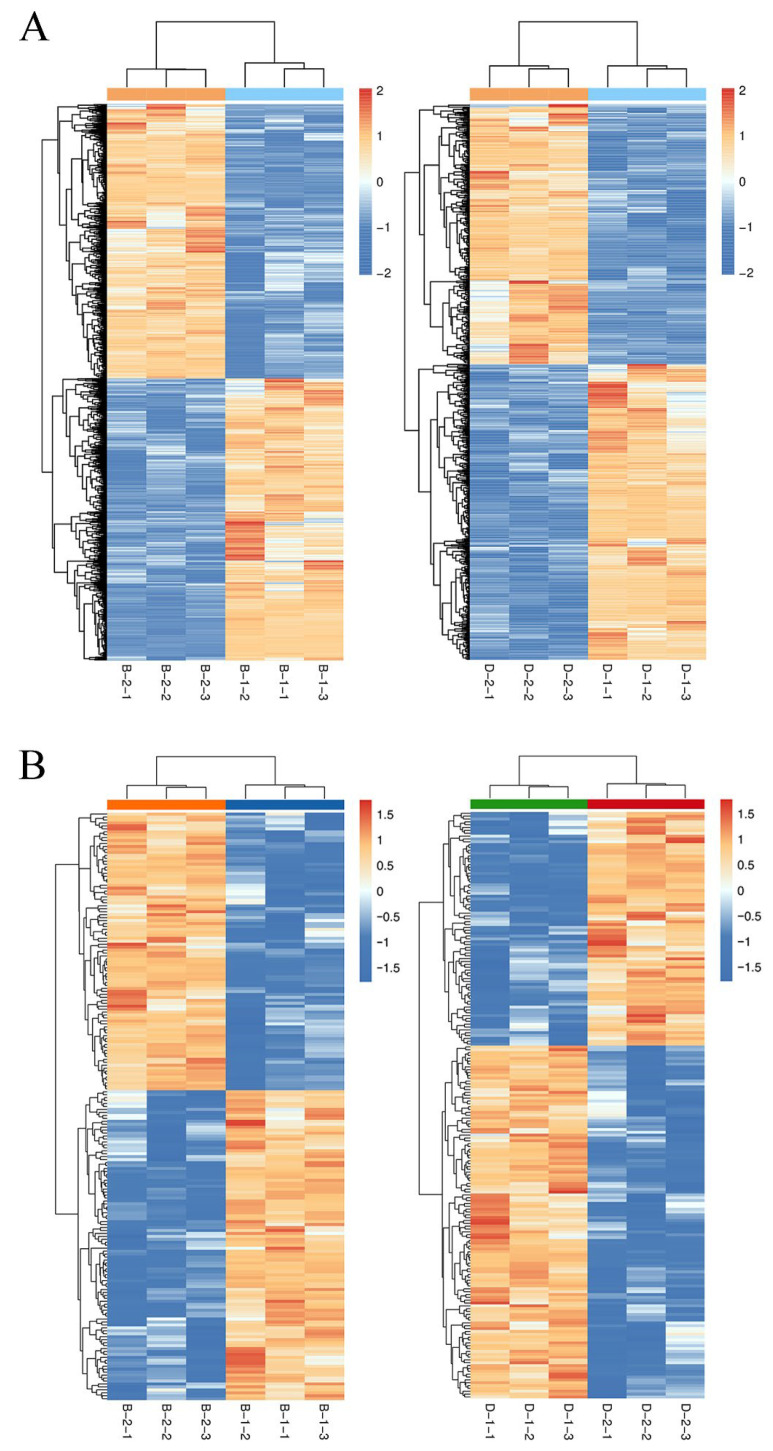
Heatmap of the DEGs (**A**) and DEPs (**B**) in the comparisons of B-1 vs. B-2 and D-1 vs. D-2 under low-salinity stress (*p* value < 0.05). Three biological replicates were performed in each group. B-1, acute stress (2 h, 8‰); B-2, acute control (2 h, 17‰); D-1, chronic stress (72 h, 8‰); and D-2, chronic control (72 h, 17‰).

Comparative multi-omics analysis revealed substantial molecular reprogramming in *S. paramamosain* megalopae under acute (2 h) and prolonged (72 h) hypoosmotic stress, with distinct differential expression patterns at both transcriptomic and proteomic levels (Table 2). RNA-seq analysis identified 2627 DEGs in acute stress comparison (B-1 vs. B-2) (Table S1; Figure 3A), with near-balanced regulation (1332 upregulated; 1295 downregulated). The top upregulated DEGs were cell wall-associated hydrolase, butyrobetaine dioxygenase, ferric-chelate reductase 1, phospholipase domain-containing protein, serine acetyltransferase, monocarboxylate transporter 9, bestrophin-3, transmembrane protein 184B and sodium-dependent nutrient amino acid transporter 1. While the top downregulated DEGs were histone-lysine N-methyltransferase E (z), serine/threonine-protein kinase stk11, carbohydrate sulfotransferase 10, galactosylceramide sulfotransferase, alpha-(1,6)-fucosyltransferase, epidermal cell surface receptor, mucin-2, calcified cuticle protein and acid phosphatase. The prolonged exposure (D-1 vs. D-2) elicited 733 DEGs (390 upregulated, 343 downregulated), representing a 72.1% reduction in responsive genes compared to acute stress. The top upregulated DEGs were zinc-binding ribosomal protein, chimeric protein, hemocyanin C chain, ferric-chelate reductase 1, peritrophin-44, proton-coupled folate transporter, tenascin, arylsulfatase H, endoglucanase E-4, annexin-B12, metallothionein and sodium/glucose cotransporter 5. As shown in Table S1, the Na^+^/K^+^/2Cl^−^ cotransporter gene was significantly upregulated under low-salinity stress (B-1 vs. B-2 and D-1 vs. D-2). Concurrently, expression of an ATP-binding cassette (ABC) transporter-related gene was significantly downregulated (B-1 vs. B-2 and D-1 vs. D-2), suggesting a potential role for these transporters in osmoregulatory processes. Moreover, the 14-3-3ζ gene was significantly downregulated under prolonged low-salinity stress (D-1 vs. D-2), whereas the crustacean hyperglycemic hormone 1 (CHH1) gene was upregulated under acute low-salinity stress (B-1 vs. B-2), indicating a rapid response involved in osmoregulation.

DIA proteomics quantified 199 DEPs under acute stress, showing slightly elevated upregulation (105 increased vs. 94 decreased) (Table S2; Figure 3B). In this comparison, the top upregulated DEPs were UBP-type domain-containing protein, TauD/TfdA-like domain-containing protein, ester hydrolase C11, anti-lipopolysaccharide factor 2, serine protease inhibitor, larval cuticle protein A3A, glycerol-3-phosphate acyltransferase 3, carbohydrate kinase PfkB domain-containing protein, retinol dehydrogenase 11, ubiquitin carboxyl-terminal hydrolase FAF-X, metallothionein-2, V-type proton ATPase subunit F, heat shock protein family member and stress-70 protein. Whereas the top downregulated DEPs were citrate synthase 1, mitochondrial import inner membrane translocase subunit, lipopolysaccharide and beta-1,3-glucan binding protein, chitin-binding type-2 domain-containing protein, endoglucanase, sphingomyelin phosphodiesterase, sterol regulatory element binding protein-1, inward rectifier potassium channel irk-1, cuticle protein CUT8, essential for reactive oxygen species protein, glutathione peroxidase and membrane metallo-endopeptidase 1. The prolonged low-salinity exposure (72 h, D-1 vs. D-2) induced 206 DEPs, with 60.2% (n = 124) upregulated and 39.8% (n = 82) downregulated. The top upregulated DEPs included rhomboid-related protein 2, RING-type E3 ubiquitin transferase, endoplasmic reticulum-based factor for assembly of V-ATPase, glycine N-acyltransferase-like protein, ubiquitin-like protein, septin-type G domain-containing protein, glutathione peroxidase, TauD/TfdA-like domain-containing protein, apolipoprotein D, uricase, endoglucanase, glycosyltransferase 25 family member, chitin-binding type-2 domain-containing protein, transporter, metalloendopeptidase, transferrin-like domain-containing protein and cuticle protein. Conversely, prominent downregulated DEPs comprised MIP18 family protein FAM96A, UBP-type domain-containing protein, vacuolar protein sorting-associated protein 13 DH-like domain-containing protein, anti-lipopolysaccharide factor, butyrobetaine dioxygenase, glutamate dehydrogenase, sodium channel protein, trypsin-like serine proteinase, cuticle protein 6, potassium voltage-gated channel subfamily H member 2, glycosyl hydrolase family 13 catalytic domain-containing protein, regucalcin and galactose-1-phosphate uridylyltransferase.

### 2.4. GO Enrichment of DEGs and DEPs

**Figure 4:**
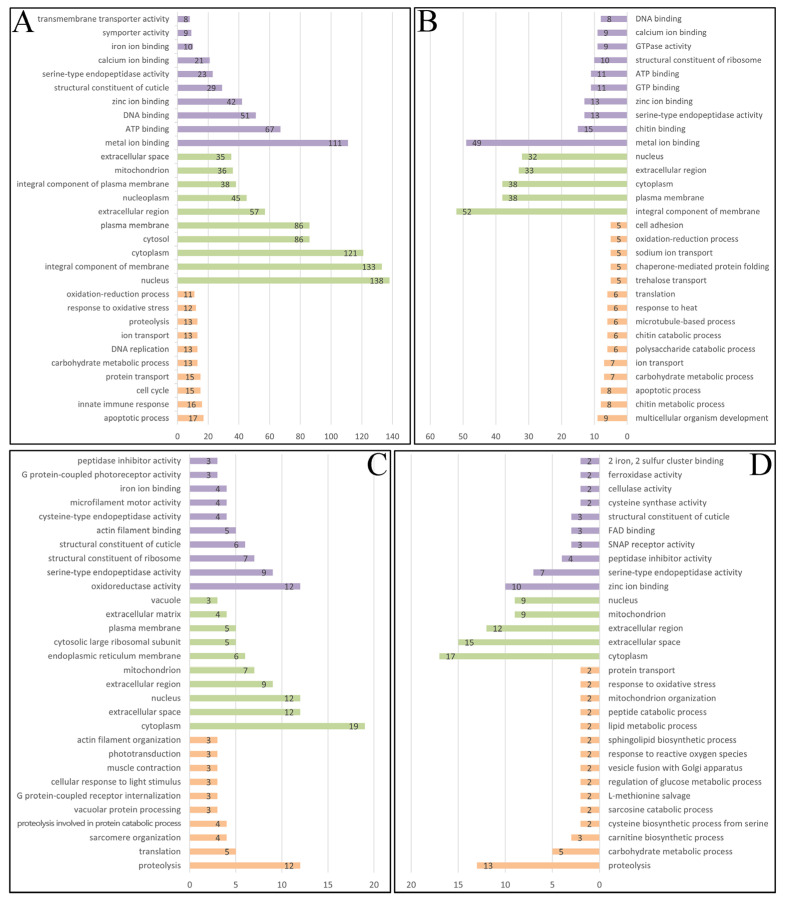
Distribution of top DEGs and DEPs among GO terms. (**A**,**B**) were the transcriptomic data comparisons of B-1 vs. B-2 group and D-1 vs. D-2 group, respectively. (**C**,**D**) were the proteomic data comparisons of B-1 vs. B-2 group and D-1 vs. D-2 group, respectively. The horizontal axis showed the hit counts of DEGs or DEPs associated with the corresponding term. Purple: molecular functions (MF); Green: cellular components (CC); Orange: biological processes (BP). B-1, acute stress (2 h, 8‰); B-2, acute control (2 h, 17‰); D-1, chronic stress (72 h, 8‰); and D-2, chronic control (72 h, 17‰).

GO enrichment analysis of acute stress-responsive DEGs (B-1 vs. B-2) revealed 1887 classifications, distributed across three ontologies: biological process (BP, 1171 terms), molecular function (MF, 435 terms) and cellular component (CC, 281 terms). The predominant BP categories (Figure 4A) included apoptotic process (GO:0006915), innate immune response (GO:0045087), cell cycle (GO:0007049), protein transport (GO:0015031), carbohydrate metabolic process (GO:0005975), DNA replication (GO:0006260), ion transport (GO:0006811), proteolysis (GO:0006508), response to oxidative stress (GO:0006979) and oxidation-reduction process (GO:0055114), reflecting core stress adaptation mechanisms. In the CC analysis, nucleus (GO:0005634), integral component of membrane (GO:0016021), and cytoplasm (GO:0005737) were the top three items. While metal ion binding (GO:0046872), ATP binding (GO:0005524), DNA binding (GO:0003677), zinc ion binding (GO:0008270), structural constituent of cuticle (GO:0042302), serine-type endopeptidase activity (GO:0004252), calcium ion binding (GO:0005509) and iron ion binding (GO:0005506) were the most significantly enriched terms in the MF.

Prolonged low-salinity exposure (D-1 vs. D-2) yielded 791 significantly enriched GO terms from DEGs (FDR < 0.05), with ontological distribution as follows: 429 BP, 210 MF and 152 CC. Figure 4B illustrated the predominant BP terms, including multicellular organism development (GO:0007275), chitin metabolic process (GO:0006030), apoptotic process (GO:0006915), carbohydrate metabolic process (GO:0005975), ion transport (GO:0006811), polysaccharide catabolic process (GO:0000272) and chitin catabolic process (GO:0006032). Whereas integral component of membrane (GO:0016021), plasma membrane (GO:0005886), cytoplasm (GO:0005737), extracellular region (GO:0005576) and nucleus (GO:0005634) were the top classifications in the CC analysis. MF enrichment was dominated by metal ion binding (GO:0046872), followed by chitin binding (GO:0008061), serine-type endopeptidase activity (GO:0004252), zinc ion binding (GO:0008270), GTP binding (GO:0005525), ATP binding (GO:0005524), structural constituent of ribosome (GO:0003735), GTPase activity (GO:0003924), calcium ion binding (GO:0005509) and DNA binding (GO:0003677).

GO enrichment for DEPs in the B-1 vs. B-2 comparison revealed 300 classifications, including 116 under the BP, 63 under the CC and 121 under MF. Among them, the most significantly enriched term in the BP was proteolysis (GO:0006508), followed by translation (GO:0006412), sarcomere organization (GO:0045214), proteolysis involved in protein catabolic process (GO:0051603) and vacuolar protein processing (GO:0006624) (Figure 4C). The top terms involved in the MF were oxidoreductase activity (GO:0016491), serine-type endopeptidase activity (GO:0004252), structural constituent of ribosome (GO:0003735), structural constituent of cuticle (GO:0042302), actin filament binding (GO:0051015) and iron ion binding (GO:0005506). Whereas in the CC, the top categories were cytoplasm (GO:0005737), extracellular space (GO:0005615), nucleus (GO:0005634), extracellular region (GO:0005576) and mitochondrion (GO:0005739).

Proteomic GO analysis of prolonged stress responses identified 374 enriched terms, with equivalent representation in BP and MF (153 terms each) and fewer CC terms (68), suggesting widespread functional reprogramming at the protein level. The most enriched term under BP category was proteolysis (GO:0006508), followed by carbohydrate metabolic process (GO:0005975), carnitine biosynthetic process (GO:0045329), cysteine biosynthetic process from serine (GO:0006535), sarcosine catabolic process (GO:1901053), regulation of glucose metabolic process (GO:0010906), response to reactive oxygen species (GO:0000302), response to oxidative stress (GO:0006979) and protein transport (GO:0015031) (Figure 4D). The top terms in the CC were cytoplasm (GO:0005737), extracellular space (GO:0005615), extracellular region (GO:0005576), mitochondrion (GO:0005739) and nucleus (GO:0005634), which were similar with those in the CC of the B-1 vs. B-2 comparison. Whereas MF category was dominated by zinc ion binding (GO:0008270), serine-type endopeptidase activity (GO:0004252), peptidase inhibitor activity (GO:0030414), structural constituent of cuticle (GO:0042302), cellulase activity (GO:0008810), ferroxidase activity (GO:0004322) and 2 iron and 2 sulfur cluster binding (GO:0051537).

### 2.5. KEGG Pathway Enrichment of DEGs and DEPs

**Figure 5:**
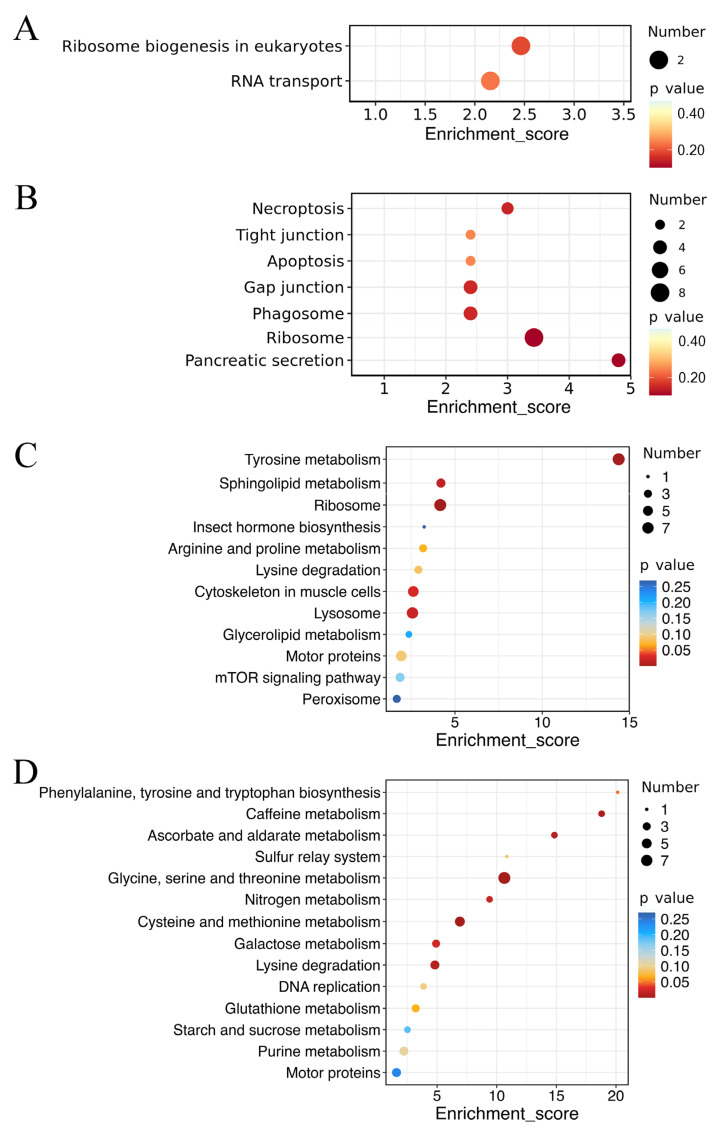
KEGG enrichment analyses of differentially expressed genes (DEGs) and proteins (DEPs) under acute and chronic low-salinity stress. The X axis represented the enrichment score, while the Y axis represented the functional pathways of KEGG. (**A**) DEGs in acute stress vs. acute control (B-1 vs. B-2, transcriptome); (**B**) DEGs in chronic stress vs. chronic control (D-1 vs. D-2, transcriptome); (**C**) DEPs in acute stress vs. acute control (B-1 vs. B-2, proteome); and (**D**) DEPs in chronic stress vs. chronic control (D-1 vs. D-2, proteome). Proteomic KEGG analysis revealed broader pathway enrichment, indicating extensive post-transcriptional regulation. Acute stress DEPs (B-1 vs. B-2) were enriched in tyrosine metabolism (esn00350), ribosome (esn03010), sphingolipid metabolism (esn00600), lysosome (esn04142) and cytoskeleton in muscle cells (esn04820), suggesting immediate metabolic shifts and cytoskeletal remodeling critical for maintaining cellular stability (**C**). Prolonged exposure (the comparison of D-1 vs. D-2) revealed that DEPs were significantly enriched in the pathways of glycine, serine, and threonine metabolism (esn00260), cysteine and methionine metabolism (esn00270), caffeine metabolism (esn00232), ascorbate and aldarate metabolism (esn00053), nitrogen metabolism (esn00910), galactose metabolism (esn00052) and lysine degradation (esn00310) (**D**), highlighting persistent amino acid metabolic adjustments and protein processing as essential strategies for chronic low-salinity adaptation.

Acute stress DEGs (B-1 vs. B-2) showed predominant enrichment in ribosome biogenesis in eukaryotes (ko03008) and RNA transport (ko03013) (Figure 5A), suggesting immediate translational reprogramming. While prolonged stress DEGs (D-1 vs. D-2) were enriched in necroptosis (ko04217), tight junction (ko04530), apoptosis (ko04210), gap junction (ko04540), phagosome (ko04145), ribosome (ko03010) and pancreatic secretion (ko04972) (Figure 5B), reflecting long-term adaptive remodeling.

### 2.6. Integrated Analysis of Transcriptome-Proteome

Multi-omics integration identified consistent DEGs/DEPs between transcriptomic and proteomic datasets (Table 3). In the comparison of B-1 vs. B-2, 18 genes exhibited significant regulation at both the transcript and protein levels. Except for cuticle protein AMP16.5 downregulated at transcriptional level and upregulated at translational level, consistent expression patterns for serine protease/proteinase, chymotrypsin, anti-lipopolysaccharide factor 1, hemocyanin, carboxylic ester hydrolase 4/6, type I crustin3/6/7, nitric oxide synthase, DNA replication licensing factor MCM4, cuticle protein 18.6, histone H5, carboxypeptidase B and chitin binding protein type 2, reflected rapid, coordinated regulatory mechanisms under acute low-salinity stress, versus only 5 gene-protein pairs (duplex-specific nuclease, lectin 3, type I crustin 3, digestive cysteine proteinase 3 and trypsin) in prolonged stress, indicating temporal regulatory divergence.

## 3. Discussion

While *S. paramamosain* possesses notable euryhaline capacity, rapid salinity reductions can induce significant physiological stress, especially during the megalopa stage when transitioning from high to low salinity environments. Omics technologies, along with the integration and comparison of various omics, can help uncover more intricate regulatory mechanisms across multiple regulatory layers [21,22]. Through conjoint transcriptome-proteome analysis, we systematically characterized temporal response patterns of *S. paramamosain* megalopa to both acute (2 h) and prolonged (72 h) hypoosmotic stress (8‰), revealing stage-specific adaptation strategies. Aligning with crustacean multi-omics studies [9], we observed greater magnitude of transcriptomic (3360 DEGs) versus proteomic (405 DEPs) changes during low-salinity stress (Table 2), confirming that mRNA abundance fluctuations often precede and exceed corresponding protein-level adjustments. This expression-level discordance underscored the crucial role of post-transcriptional regulation and post-translational modifications in mediating physiological adaptation under environmental stress.

Low-salinity stress disrupts osmotic balance in euryhaline crabs, requiring rapid regulation of ion transport genes, owing to ion transporters serving as key component of osmoregulation [9,23]. Acute hypoosmotic challenge (2 h) induced significant differential expression of 13 ion transport-related genes, such as Na^+^-K^+^-2Cl^−^ cotransporter, ABC transporter, transferrin, cation transporter, sodium-coupled monocarboxylate transporter 1, sodium-independent sulfate anion transporter and transient receptor. As a 48-member superfamily of membrane proteins, ABC transporters actively transport various biological substrates across lipid membranes, and are involved in the absorption of small molecules and in the regulation of membrane ion channels [11,24]. The ABC transporter gene differentially expressed in the current study (top 100 in Table S1) was most likely to be involved in osmoregulation. However, under the prolonged low-salinity stress, ABC transporter did not appear in the list (top 100 in Table S1) of significant DEGs in our study, suggesting that the mud crab megalopa may adopt distinct ion transport regulation strategies to adapt to acute and prolonged low-salinity changes. For example, sodium ion transport, including the unigenes of solute carrier family 23 member 1, sodium/glucose cotransporter 5, ileal sodium/bile acid cotransporter and sodium-coupled monocarboxylate transporter 1, was significantly enriched in GO terms, whereas it was absent during the acute low-salinity response. Na^+^-K^+^-2Cl^−^ cotransporter plays essential role in osmotic regulation and cell ionic via transporting Na^+^, K^+^ and 2Cl^−^ into cells [25]. Previous studies showed that the transcription profiles of Na^+^-K^+^-2Cl^−^ cotransporter were significantly affected by salinity change [26]. In the present study, the Na^+^-K^+^-2Cl^−^ cotransporter was upregulated in *S. paramamosain* megalopa subjected to both acute and prolonged low-salinity stress. This result contrasted with a previous report that documented downregulated expression of Na^+^-K^+^-2Cl^−^ cotransporter under low salinity in *S. paramamosain* megalopa [11], further demonstrated that the mechanisms underlying ion transport gene-mediated osmoregulation in the megalopa could be highly complex and dynamic, varying across different developmental stages and salinity conditions. Besides the ion transport, osmotic signal transduction is also important in the molecular mechanisms of osmoregulation, but it is seldom explored in crustaceans [9]. The evolutionarily conserved 14-3-3 protein family has emerged as a critical player in osmoregulation through interaction with key ion transporters and pathways [27]. The expression patterns of 14-3-3 exhibited a trend similar to Na^+^/K^+^-ATPase activity in *Litopenaeus vannamei* under low-salinity stress, suggesting that 14-3-3 plays a critical role in the regulatory network controlling Na^+^/K^+^-ATPase function [27]. In *Penaeus monodon*, 14-3-3B mRNA significantly decreased under hyperosmotic stress but increased upon hypoosmotic challenge, indicating its involvement in osmotic regulation [28]. We found that the expression level of 14-3-3ζ was significantly downregulated only under the prolonged low-salinity stress, indicating its lower sensitivity to salinity changes. In addition, we identified crustacean hyperglycemic hormone 1 (CHH1), another candidate gene involved in osmotic signal transduction. The CHH, a neuropeptide produced primarily in the X-organ/sinus gland (XO-SG) complex of the eyestalk and other neuroendocrine tissues, is a key hormone in low salinity acclimatization in decapod crustacean [29]. In contrast to the 14-3-3 gene expression profile, CHH1 in this study was upregulated under acute low salinity stress, supporting its quick response to participate in osmoregulatory signal transduction during early stage of low-salinity adaptation in *S. paramamosain*.

Osmoregulation under hypoosmotic conditions imposes substantial energetic demands on crustaceans, requiring enhanced metabolic activity to sustain iono- and osmoregulatory processes [9,30]. Multi-omics analysis revealed significant enrichment of energy-generating pathways, including carbohydrate metabolism, chitin catabolism, polysaccharide catabolism, chitin metabolism, proteolysis, peptide catabolism, lipid metabolism and amino acid catabolism (Figure 4), indicating metabolic reprogramming to meet the elevated ATP demands of *S. paramamosain* megalopa under low-salinity stress.

Within carbohydrate metabolic pathways, alpha-amylase exhibited the most pronounced differential expression in the current study. As an important glucose glycosidase enzyme, amylase plays a crucial role in carbohydrate digestion [31]. In crustaceans, amylase activity strongly correlated with hepatopancreatic digestive efficiency and nutrient assimilation rates [32]. Environmental salinity fluctuations are known to modulate digestive enzyme activity in euryhaline species, thus affecting relative metabolic functions [33]. In this study, alpha-amylase was significantly upregulated under low-salinity conditions, suggesting enhanced carbohydrate catabolic capacity. Amino acid metabolism is integral for maintaining osmotic balance and supporting cellular repair mechanisms under osmotic stress [34]. Notably, acute hypoosmotic stress (2 h, 8‰) specifically induced tyrosine metabolism. Whereas prolonged low-salinity exposure (72 h) activated broader amino acid metabolism, such as phenylalanine, tyrosine and tryptophan biosynthesis, glycine, serine and threonine metabolism, cysteine and methionine metabolism, and lysine degradation (Figure 5), suggesting stage-specific metabolic adaptation where amino acids potentially took on responsibility for providing energy and sustaining cellular homeostasis. Our observations contrasted with prior findings in *S. paramamosain*, where amino acid metabolic pathways showed minimal enrichment in response to low-salinity stress [9]. This discrepancy likely reflected ontogenetic differences in osmoregulatory strategies of mud crabs. Collectively, these results demonstrated developmental stage-specific modulation of amino acid metabolism within osmoregulatory networks, with megalopa stage exhibiting greater metabolic plasticity than the following developmental stages.

In marine invertebrates, alterations in environmental salinity could induce multiple physiological responses, particularly oxidative stress, perturbing the maintenance of cell homeostasis [35,36]. When the homeostasis is disrupted, oxidative stress can lead to abnormal cell death, or even promote disease development [37]. Our results revealed significant enrichment of DEGs/DEPs in key oxidative stress-related GO terms (Figure 4), including oxidation-reduction process, response to oxidative stress, apoptosis, response to heat, response to reactive oxygen species, phagosome and lysosome, demonstrating coordinated stress response networks. Moreover, enrichment in proteolysis in this study, highlighted active protein turnover regulation, likely critical for maintaining cellular homeostasis under salinity stress [38]. Additionally, enrichment in extracellular space and extracellular region implied potential modifications of extracellular matrix components, potentially impacting osmoregulatory mechanisms [39]. The structural constituent of cuticle (GO:0042302) exhibited significant changes in expression levels under low-salinity stress, with the expression levels of several cuticle-related proteins being increased and others decreased, suggesting potential exoskeletal remodeling as part of the adaptive response [9]. Therefore, the relative enrichment of pathways further underlined proteins as crucial in oxidative stress defense under low-salinity conditions [40]. These findings suggested that exposure to low-salinity stress could disrupt cell homeostasis, induce reactive oxygen species (ROS) imbalance, and subsequently lead to oxidative stress in *S. paramamosain* megalopa.

The crustacean innate immune system serves as the primary defense mechanism against environmental stressors [41]. Environmental factors have been shown to induce fluctuations in hemocyanin concentration, particularly exhibiting elevated levels in crustaceans under low-salinity conditions, implying that the molecular heterogeneity of hemocyanin possesses adaptive plasticity to environmental challenges [42]. Crustacean hemocyanin, composed of multiple subunits and exhibiting flexible expression, is essential for modulating oxygen transport, which might be affected by the salinity change [11,42]. Moreover, hemocyanin plays an important role in non-specific innate immune defense and is an effective immune defense molecule in arthropods [43]. Two significantly differentially expressed hemocyanin transcripts, hemocyanin subunit 4 and hemocyanin C chain, were found in this study. The upregulated hemocyanin C chain in the D-1 group indicated its participation in the prolonged low-salinity response. However, hemocyanin subunit 4 at both transcriptional and proteinic levels were downregulated, potentially reflecting metabolic trade-offs between oxygen transport and immune functions during prolonged stress adaptation that warrant further investigation. Lectins, involved in innate immunity, are important pattern recognition receptors in animals, plants, and microorganisms. They specifically bind to microbial surface carbohydrates, triggering immune responses that eliminate invading pathogens [44], and playing important roles in various environmental stresses [45]. A prior study demonstrated that its expression was found to be slightly increased followed by salinity changed in turbot gill [46]. In the present study, the expression of lectin 3 was significantly upregulated at both transcriptional and proteinic levels. We speculated that it might be involved in the low-salinity stress response, potentially through interactions with carbohydrate components from significantly enriched processes such as carbohydrate metabolism identified in this study. While the underlying mechanisms merit further study. As multi-domain cysteine-rich antimicrobial polypeptides (AMPs), crustins appear to have an important function in the innate immune system. The malacostracan crustins comprise four phylogenetically distinct types (I–IV), classified according to variations in their N-terminal structural domains [47]. Accumulating evidence indicates that crustins, beyond their well-characterized antimicrobial functions, are critically involved in mediating stress responses. For instance, the transcript levels of a crustin gene in epipodite were increased under salinity stress in *P. monodon* [48]. In the present study (Table 3), crustins, including crustin 3, crustin 6 and crustin 7, were observed to be upregulated at both transcriptional and proteinic levels under the acute hyposaline exposure. Conversely, prolonged exposure specifically downregulated crustin 3, demonstrating isoform-specific temporal regulation. These data indicated that crustins were functionally involved in physiological low-salinity response in *S. paramamosain* megalopa. Usually, the response patterns of immune-related genes shift between acute and prolonged stress phases. Initially, acute stress provokes immune activation, potentially as a generalized stress reaction, whereas prolonged stress leads to a downregulation of certain immune effectors. This immune modulation pattern, initial activation followed by suppression, is frequently observed in crustaceans exposed to prolonged environmental stressors [13,35].

Notably, acute hypoosmotic stress induced significant dysregulation of DNA replication in the megalopa (Figure 4). Enhanced expression of genes related to DNA replication indicated potential regulation of cell-cycle progression, impacting cell proliferation and genomic stability under stress conditions [9,35]. Prolonged exposure to low salinity appeared to promote adaptive mechanisms through structural remodeling of the exoskeleton. Significant enrichment of genes related to chitin metabolism and catabolism in our study indicated alterations in exoskeletal composition, enhancing long-term resilience to osmotic stress [12].

Our integrated multi-omics analysis revealed limited concordance between DEGs and DEPs, with only 22 common genes showing consistent expression changes at both transcriptional and translational levels (Table 3). This weak transcript-protein correlation reflects the intricate multi-layer regulation involved in environmental adaptation processes. Similar findings have been reported in crustaceans, particularly during salinity adaptation in mud crabs [9,49]. These observed discrepancies might originate from comprehensive regulatory mechanisms encompassing post-transcriptional modifications, translational control and protein degradation pathways, which collectively modulated protein abundance independently of transcriptional changes. Although the correlation was low, the complementary information derived from both transcriptomic and proteomic datasets enabled a more holistic understanding of salinity adaptation mechanisms. Transcriptomic profiling identified significant alterations in genes related to apoptotic process, innate immune response, cell cycle, DNA replication, carbohydrate metabolism and ion transport. Conversely, proteomic analysis detected substantial changes in protein abundance associated with translation, proteolysis, as well as carbohydrate, lipid, and amino acid metabolic pathways, with particular emphasis on the crucial role of carbohydrate metabolism in osmoregulatory adaptation. Consequently, our multi-omics integration strategy provided unique and valuable insights into the molecular basis of environmental adaptation in crustaceans. The *S. paramamosain* megalopa possibly maintained its low-salinity adaptation by regulating the DEGs and DEPs associated with these pathways, exhibiting an inherent capacity for salinity stress resistance that might be closely related to its life history characteristics [11]. These findings suggested that the megalopa stage could serve as a promising target for selective breeding programs of low-salinity-tolerant mud crabs, thereby facilitating the sustainable expansion of *S. paramamosain* aquaculture practices.

## 4. Materials and Methods

### 4.1. Salinity Challenge and Samples Collection

The salinity challenge experiments were performed at the Ninghai Research Center of the East China Sea Fisheries Research Institute, Chinese Academy of Fishery Sciences, Zhejiang Province, China. Megalopae were maintained in 80-L polyethylene tanks under controlled conditions: 17‰ salinity, ~26 °C water temperature and >7 mg/L dissolved oxygen (DO), with daily feeding of *Artemia* nauplii [20]. Healthy *S. paramamosain* megalopae exhibiting normal morphology and vigorous swimming behavior at one day post-metamorphosis from the zoea stage were selected and maintained in another experimental 80-L white tanks. For acute exposure (B-1 group), 500 megalopae were subjected to 8‰ salinity for 2 h, and prolonged exposure (D-1 group) involved 72 h was cultured at 8‰ salinity. Control groups (B-2 and D-2) were maintained at 17‰ salinity for 2 h and 72 h, respectively. Post-treatment, 30 individuals per group were collected and pooled (10 individuals/replicate) to generate three biological replicates. Samples were flash-frozen in liquid nitrogen and stored at −80 °C until RNA extraction.

### 4.2. Transcriptomic Analysis

Total RNA extraction was performed using TRIzol Reagent (Invitrogen, Waltham, MA, USA) according to the manufacturer’s instructions. RNA quality was evaluated using both the Agilent 2100 Bioanalyzer (Agilent Technologies, Santa Clara, CA, USA) for RNA integrity number (RIN) assessment and Nanodrop 2000 spectrophotometer (Thermo Fisher Scientific, Waltham, MA, USA) for purity determination. Only RNA samples meeting stringent quality criteria (A_260/280_ ratio 1.8–2.2; RIN ≥ 9) were processed for library construction. RNA-seq libraries were prepared using the VAHTS Universal V6 RNA-seq Library Prep Kit (Vazyme, Nanjing, China) and sequenced on an Illumina NovaSeq 6000 platform (Illumina, San Diego, CA, USA). High-throughput sequencing was performed by OE Biotech Co., Ltd. (Shanghai, China) to generate 150 bp paired-end reads. Raw sequencing reads were quality-filtered using Trimmomatic v0.39 [50] to eliminate adapter sequences and low-quality bases. De novo transcriptome assembly was performed using Trinity v2.11.0 with default parameters [51]. Transcriptome completeness was evaluated using BUSCO v3.0.2 [52]. Transcript annotation was performed by BLASTX (part of BLAST+ package, version 2.13.0) searches against the NCBI non-redundant protein database (E-value cutoff 1 × 10^5^). Gene identification was based on top BLAST hits (bit score > 50) to known sequences. Gene expression levels were quantified as fragments per kilobase per million mapped reads. Differential gene expression analysis was performed using the Trinity for comparison among groups with three replicates. Differentially expressed genes (DEGs) were identified using DESeq2 with a significance threshold of *p*-value < 0.05 and foldchange > 2 or <−2 [53]. Principal component analysis (PCA) was conducted using variance-stabilized transformed counts from DESeq2 to assess overall transcriptome variation.

### 4.3. Proteomic Analysis

Total proteins were extracted from homogenized megalopa tissues in lysis buffer (8 M urea and 1% protease inhibitors). After centrifugation (12,000× *g*, 15 min, 4 °C), protein concentration was determined using the Pierce BCA Protein Assay Kit (Thermo Scientific, Waltham, MA, USA) with bovine serum albumin as standard. Protein samples were reduced with 5 mM dithiothreitol, alkylated with 15 mM iodoacetamide, and digested with sequencing-grade trypsin at a 1:50 enzyme-to-protein ratio overnight. Resulting peptides were labeled with tandem mass tags and fractionated by high-pH reverse-phase chromatography to reduce complexity.

Labeled peptides were analyzed using a Thermo Scientific LC-MS/MS system. Raw MS files were analyzed using Proteome Discoverer v2.4 (Thermo Scientific) against the UniProt *S. paramamosain* database. Protein identification and LFQ quantification were performed using MaxQuant v1.6.5.0 [54] with carbamidomethylation as fixed modification and oxidation (M), acetyl (N-term) as variable modifications. Protein quantification was normalized based on peptide-spectrum match counts. Differentially expressed proteins (DEPs) were defined by significance thresholds of *p*-value < 0.05 and fold change ≥ 1.2 or ≤0.83. PCA was applied to the processed matrix using ‘prcomp’ (scaling enabled) to assess global proteomic variation. Transcript-protein correlation analysis conducted using paired TPM (RNA-seq) and iBAQ (proteomics) values after log2 transformation.

### 4.4. Functional Annotation and Statistical Analysis

Functional annotation of genes was performed through GO classification using Blast2GO v5.2 and KEGG pathway mapping [55]. Enrichment analysis was conducted with clusterProfiler v3.10.1, with *p*-value < 0.05 as the significant threshold. Differential expression patterns were compared between two experimental conditions: (1) acute hypoosmotic stress (8‰ for 2 h; B-1 vs. control B-2) and (2) prolonged hypoosmotic exposure (8‰ for 72 h; D-1 vs. control D-2). Statistical significance was determined using false discovery rate (FDR) correction (q < 0.05) for transcriptomic data and two-tailed Student’s *t*-tests (*p* < 0.05) for proteomic results.

## 5. Conclusions

This study employed an integrated transcriptomic and proteomic approach to elucidate the molecular mechanisms underlying acute and prolonged low-salinity adaptation in the megalopa stage of *S. paramamosain*. Low-salinity stress triggered significant alteration of ion transporters (e.g., Na^+^-K^+^-2Cl^−^ cotransporter, ABC transporter and transferrin), osmotic signal transduction (e.g., 14-3-3 and crustacean hyperglycemic hormone 1), energy metabolism (e.g., carbohydrate and amino acid metabolism) and immune response (e.g., hemocyanin, crustins and lectins). Furthermore, acute low-salinity exposure substantially disrupted DNA replication and cell-cycle progression, potentially compromising cell proliferation and genomic stability during stress conditions. Conversely, prolonged stress stimulated adaptive exoskeletal remodeling through chitin metabolic reorganization, suggesting structural modifications that may enhance osmotic stress resistance during sustained exposure. Both omics datasets revealed significant enrichment of energy metabolism-related pathways, indicating potential upregulation of metabolic activity in *S. paramamosain* megalopa to meet heightened energy demands during low-salinity stress. Collectively, this study substantially advanced our comprehension of salinity adaptation mechanisms by systematically characterizing specific molecular pathways and potential regulatory strategies employed by *S. paramamosain* megalopa. The integrated multi-omics data will serve selective breeding programs aimed at enhancing low-salinity tolerance in *S. paramamosain* aquaculture.

## Figures and Tables

**Table 1:** Transcriptome sequencing results for *S. paramamosain* megalopae.

Sample	Raw Reads (M)	Raw Bases (G)	Clean Reads (M)	Clean Bases (G)	Valid Bases (%)	Q30 (%)	GC (%)
B-1-2	48.37	7.13	47	6.92	97.17	97	53.31
B-1-2	44.78	6.59	43.42	6.39	96.95	96.95	53.89
B-1-3	48.28	7.1	46.79	6.88	96.92	96.94	53.33
B-2-1	47.41	7	46.19	6.82	97.42	97	52.74
B-2-2	48.07	7.08	46.66	6.87	97.07	97.01	52.44
B-2-3	48.23	7.13	47.05	6.96	97.55	97.13	52.58
D-1-1	43.75	6.45	42.52	6.27	97.19	96.92	52.27
D-1-2	49.57	7.28	47.94	7.04	96.72	96.82	52.75
D-1-3	50.61	7.34	48.41	7.02	95.65	96.91	54.07
D-2-1	46.18	6.79	44.72	6.57	96.83	96.91	53.67
D-2-2	48.66	7.16	47.19	6.94	96.97	97.02	52.89
D-2-3	49.34	7.26	47.78	7.03	96.84	96.83	52.36

Note: three biological replicates were performed in each group. B-1, acute stress (2 h, 8‰); B-2, acute control (2 h, 17‰); D-1, chronic stress (72 h, 8‰); and D-2, chronic control (72 h, 17‰).

**Table 2:** Comparison of differentially expressed genes (DEGs) and proteins (DEPs).

Comparison Group	Upregulated DEGs	Downregulated DEGs	Upregulated DEPs	Downregulated DEPs
B-1 vs. B-2	1332	1295	105	94
D-1 vs. D-2	390	343	124	82

Note: B-1, acute stress (2 h, 8‰); B-2, acute control (2 h, 17‰); D-1, chronic stress (72 h, 8‰); and D-2, chronic control (72 h, 17‰).

**Table 3:** Integrative analysis of consistent DEGs/DEPs at both transcriptomic and proteomic levels.

	UniProt ID	DEGs			DEPs			Annotation
		log2FC	*p*-Value	Regulation	log2FC	*p*-Value	Regulation	
B-1 vs. B-2	Q2V6T8	−2.55	4.94 × 10^−170^	Down	0.24	0.0056	Up	cuticle protein AMP16.5
	D2DST4	1.10	5.64 × 10^−70^	Up	0.62	0.0053	Up	serine protease
	D6N3A2	1.38	1.07 × 10^−95^	Up	0.52	0.00089	Up	serine proteinase
	F8UN04	−1.11	1.91 × 10^−78^	Down	−0.34	0.0044	Down	chymotrypsin
	H6ACV4	1.29	7.72 × 10^−77^	Up	0.43	0.0073	Up	clip domain serine proteinase 1
	I1VGP3	1.70	1.99 × 10^−110^	Up	1.64	0.00029	Up	anti-lipopolysaccharide factor 1
	A0A0U1ZZP8	−1.03	5.06 × 10^−47^	Down	−0.67	0.00065	Down	Hemocyanin subunit 4
	A0A3S5XFQ9	−1.16	2.00 × 10^−8^	Down	−0.44	0.00091	Down	carboxylic ester hydrolase 4
	A0A3S5WLH8	1.20	7.40 × 10^−17^	Up	0.46	0.0012	Up	carboxylic ester hydrolase 6
	A0A343T7I5	1.17	1.92 × 10^−44^	Up	1.045	0.0032	Up	type I crustin 6
	A0A2K9UW22	1.36	1.65 × 10^−40^	Up	1.97	0.0040	Up	type I crustin 3
	G0M6G3	1.39	4.41 × 10^−55^	Up	0.24	0.037	Up	nitric oxide synthase
	A0A5B7CRZ2	−1.36	3.32 × 10^−27^	Down	−0.15	0.011	Down	DNA replication licensing factor MCM4
	A0A5B7DHC6	−1.78	9.16 × 10^−48^	Down	0.14	0.012	Up	cuticle protein 18.6
	A0A5B7FPJ8	−1.55	6.47 × 10^−52^	Down	−0.38	0.044	Down	chitin binding protein type-2
	A0A5B7GC89	1.46	5.43 × 10^−117^	Up	0.23	0.029	Up	histone H5
	A0A6G9W3K2	−1.66	1.45 × 10^−106^	Down	−0.61	0.0055	Down	carboxypeptidase B
	A0A858Z4L6	1.04	3.67 × 10^−40^	Up	1.98	3.63 × 10^−6^	Up	type I crustin 7
D-1 vs. D-2	K9LU24	1.32	1.16 × 10^−11^	Up	0.74	0.0036	Up	duplex-specific nuclease
	A0A068BC87	1.26	2.48 × 10^−30^	Up	0.71	0.0044	Up	lectin 3
	A0A2K9UW22	−1.99	2.93 × 10^−37^	Down	−0.92	0.00050	Down	type I crustin 3
	A0A5B7E7D2	1.41	2.01 × 10^−37^	Up	0.42	0.022	Up	digestive cysteine proteinase 3
	A0A6G9W3Z8	1.04	1.61 × 10^−26^	Up	−1.09	4.76 × 10^−5^	Down	trypsin

Note: B-1, acute stress (2 h, 8‰); B-2, acute control (2 h, 17‰); D-1, chronic stress (72 h, 8‰); and D-2, chronic control (72 h, 17‰).

## Data Availability

The datasets for this study can be found in the NCBI SRA Sequence Database at http://www.ncbi.nlm.nih.gov/bioproject/1291268 (accessed on 29 August 2025), under reference number PRJNA1291268, and in the iProx Database at https://www.iprox.cn/page/project.html?id=IPX0012647000 (accessed on 29 August 2025), under reference number PXD066248, respectively.

## Supplementary Materials

The following supporting information can be downloaded at: https://www.mdpi.com/article/10.3390/ijms26189188/s1.
